# pH-dependent effects of pepsin and trypsin on the stability and antibiofilm functionality of pea protein–stabilized carvacrol nanoemulsions

**DOI:** 10.1016/j.fochx.2025.103450

**Published:** 2025-12-26

**Authors:** Jun Ji, Mohamed Brahmi, Emilie Dumas, Nour-Eddine Chihib, Adem Gharsallaoui

**Affiliations:** a Univ. Lyon, Université Claude Bernard Lyon 1, CNRS, LAGEPP UMR 5007, 43 Bd 11 Novembre 1918, 69622 Villeurbanne, France; b University of Lille, CNRS, INRAE, Centrale Lille, UMR 8207-Unité Matériaux et Transformations, 59120 Lille, France

**Keywords:** Pea protein isolate, Nanoemulsion, Trypsin, Carvacrol, pH-dependent stability, Antibiofilm activity, Molecular docking, Food-grade antimicrobial system

## Abstract

This study investigated how proteolytic enzymes influence the physicochemical stability and bioactivity of pea protein isolate (PPI)–stabilized nanoemulsions encapsulating carvacrol at pH 3.5, 7.0, and 10.0. Pepsin or trypsin (0.1 wt%) was incorporated into pre-formed nanoemulsions (1 wt% PPI, 5 wt% carvacrol) to evaluate pH-dependent effects on structure and function. Under neutral and alkaline conditions, nanoemulsions showed submicron droplets (≈180–254 nm), high ζ-potentials (−26.5 to −41.9 mV), and excellent stability (4 weeks, CI < 5 %). Trypsin introduction enhanced emulsion stability through moderate interfacial hydrolysis, while pepsin caused destabilization under acidic conditions (≈1.5 μm, PDI ≈ 1.0). Carvacrol determined antibacterial activity (MIC = 312.5 μg/mL), whereas antibiofilm performance was pH- and enzyme-dependent: trypsin markedly enhanced biofilm eradication at pH 7.0–10.0, and pepsin had limited effect. Molecular docking revealed strong electrostatic pepsin–protein interactions causing destabilization, while trypsin bound non-catalytically, reinforcing interfacial cohesion.

## Highlights

• Pepsin and trypsin were incorporated into pea protein-stabilized nanoemulsions.
• Nanoemulsion stability was strongly governed by pH and enzyme type.
• Trypsin improved interfacial stability and enhanced antibiofilm performance.
• Pepsin induced emulsion destabilization under acidic conditions.
• Molecular docking clarified enzyme–protein interactions driving emulsion behavior.

## 1 Introduction

*Listeria monocytogenes* is a high-priority foodborne pathogen renowned for its persistence on food-contact surfaces, tolerance to sanitation procedures, and ability to cross-contaminate ready-to-eat products. The non-pathogenic surrogate *Listeria innocua* is widely employed to model *L. monocytogenes* in laboratory and pilot-scale studies owing to their comparable ecological and surface behaviors (Mohan et al., 2019). Critically, *Listeria* readily forms robust biofilms composed of a viscoelastic extracellular polymeric substance (EPS) matrix containing polysaccharides, proteins, and extracellular DNA, on stainless steel, rubber, and plastic substrates commonly found in food-processing environments. This EPS network retains nutrients, buffers pH and oxidative stress, and hinders antimicrobial diffusion, thereby conferring tolerance levels far exceeding those of planktonic cells (Damyanova & Paunova-Krasteva, 2025). Persistent *Listeria* biofilms represent a major risk for both the food industry and healthcare sectors, motivating the development of strategies that can penetrate and dismantle the EPS matrix while ensuring bactericidal efficacy under food-safe and clean-label conditions.

Conventional sanitation approaches (chemical disinfectants such as chlorine, peracetic acid, and quaternary ammonium compounds, or physical methods including pulsed light, UV irradiation, and steam) remain the backbone of hygiene programs (Hua & Zhu, 2024). However, their effectiveness is often restricted by odor and residue issues, biofilm resistance, and the need for high concentrations or prolonged contact times. These limitations have spurred growing interest in biologically inspired strategies. Among them, proteolytic enzymes are particularly attractive as they can cleave proteinaceous scaffolds within the EPS and compromise biofilm integrity. Pepsin and trypsin, for instance, have been shown to markedly degrade *Pseudomonas aeruginosa* and *Enterococcus faecalis* biofilms after short exposures (Mechmechani et al., 2023). Nevertheless, proteases alone lack bactericidal activity and often result in partial biofilm removal, potentially releasing free bacterial cells capable of recolonization. Consequently, the co-delivery of proteases with potent antimicrobials is a promising strategy, provided that an appropriate carrier system ensures co-localization, controlled release, and maintenance of enzyme functionality.

Plant essential oils (EOs) represent clean-label antimicrobials consistent with consumer and regulatory trends. For example, Kim et al. (2023) reported that DNase I combined with eugenol significantly reduced *Salmonella* biofilms on smoked duck, while the sequential use of enzymes with Aleppo pine EO decreased *S. enterica* biofilms on stainless steel by 4.6 log CFU/coupon (Olaimat et al., 2024). Among EOs, carvacrol, a phenolic monoterpene, exhibits strong activity against *Listeria* species. It permeabilizes bacterial membranes, disrupts the proton motive force, interferes with quorum-sensing mechanisms, and weakens EPS cohesion (Burt et al., 2014). Yet, its application is hindered by high hydrophobicity, volatility, low aqueous dispersibility, and possible sensory impacts. A rational solution lies in the formulation of plant protein–stabilized nanoemulsions for EO encapsulation, enhancing dispersibility, interfacial contact, and apparent bioavailability. The incorporation of proteases (e.g., pepsin or trypsin) into such systems may further enable synergistic delivery: enzymes dismantle the EPS matrix, while carvacrol provides rapid bactericidal action. The main challenge remains to ensure that enzyme integration does not compromise the colloidal stability of the nanoemulsion.

Recent studies have explored various vegetable proteins, such as soy, zein, and rice proteins, as natural emulsifiers for delivering EOs. Pea protein isolate (PPI) offers distinct advantages due to its balanced amino acid composition, allergen-free and gluten-free nature, less anti-nutritional factors than soy, high global acceptance and superior interfacial activity derived from its dominant globulin fractions (legumin 11S and vicilin 7S) (Olsmats & Rennie, 2024). Compared with soy or zein systems, PPI-stabilized nanoemulsions exhibited better colloidal stability across a wide pH range and are more compatible with clean-label formulations (Galani et al., 2023; Nasrabadi et al., 2025; Xie et al., 2024), making them particularly suitable for food-grade antimicrobial applications. Most previous studies have used enzymatic pre-hydrolysis or cross-linking to modify the emulsifying performance of proteins (Klost & Drusch, 2019). In contrast, the direct incorporation of active proteases into pre-formed, protein-stabilized nanoemulsions to modulate their biofunctionality, particularly antibiofilm properties, while maintaining colloidal stability remains largely unexplored. Current research trends have primarily focused on the direct or sequential application of enzymes and EOs for the eradication of biofilms formed on various biotic and abiotic surfaces, with limited attention to nanoemulsion systems. Ababneh et al. reported that sequential treatment with a combination of multiple hydrolytic enzymes for 30 min followed by Aleppo pine EO for another 30 min reduced *Cronobacter sakazakii* biofilms on stainless steel and plastic surfaces by 4.4–4.5 log CFU/coupon (Ababneh et al., 2025). In addition, Mechmechani et al. demonstrated that microencapsulated proteases and sodium caseinate-stabilized carvacrol exhibited significant synergistic effects against *P. aeruginosa* and *E. faecalis* biofilms when applied using hurdle technology (Mechmechani et al., 2023). However, to our knowledge, few studies have yet focused on the co-localization of enzymes and essential oils within plant protein-stabilized nanoemulsion systems for direct biofilm eradication. In such systems, protease incorporation could have dual effects on interfacial proteins: moderate hydrolysis may produce amphiphilic peptides that strengthen the interfacial layer and increase electrostatic repulsion, whereas excessive cleavage may erode the interfacial film, reduce steric and electrostatic stabilization, and trigger droplet aggregation. At the same time, this system could simplify future biofilm eradication formulations by enabling simultaneous delivery of enzymes and essential oils without requiring sequential or combined treatments. These effects are governed by the pH-dependent activity and specificity of proteases, and by the charge and hydrophobic patterns of legumin and vicilin at the oil–water interface. Thus, enzyme–protein interfacial interactions under varying pH conditions are likely to determine both colloidal stability and biological functionality.

At the molecular level, the mechanisms governing how pepsin and trypsin recognize, bind, and potentially hydrolyze legumin and vicilin at the interface remain poorly understood. Molecular docking provides valuable insight into such interactions, revealing potential binding sites and interaction energies between proteases and substrate proteins (Xue et al., 2016). For proteases, docking can also indicate whether substrate segments align with catalytic motifs, such as the Asp dyad in pepsin and the His-Asp-Ser triad with the anionic S1 pocket (Asp189) in trypsin, thereby predicting limited or extensive interfacial proteolysis. Integrating these in silico predictions with multiscale experimental data (physicochemical and biological measurements) may thus establish a mechanistic connection between interfacial chemistry and the macroscopic performance of emulsions.

The objective of this study was to evaluate the effect of protease incorporation (pepsin or trypsin) on the physicochemical stability and biological functionality of carvacrol-loaded PPI nanoemulsions prepared under acidic (pH 3.5), neutral (pH 7.0), and alkaline (pH 10.0) conditions. Specifically, the effects of enzyme addition on particle size distribution, ζ-potential, viscosity, creaming behavior, microstructure, and storage/thermal stability were investigated. The antibacterial and antibiofilm activities against *L. innocua* were then assessed, and protein–protein docking simulations were performed to elucidate the underlying molecular mechanisms. The findings provide new insight into designing clean-label, plant-based antimicrobial nanoemulsions with dual enzyme–EO functionality for food and healthcare sanitation applications.

## 2 Materials and methods

### 2.1 Materials

Trypsin from porcine pancreas (1000–2000 BAEE units/mg solid, EC 232–650-8) and imidazole (≥ 99.5 %) were obtained from Sigma-Aldrich (St. Louis, MO, USA). Pepsin A (≥ 2000 Hemoglobin units/mg solid, EC 232–629-3) was purchased from MP Biomedicals (Strasbourg, France). Carvacrol (≥ 98 % purity; MW = 150.22 g/mol; density = 0.976 g/cm^3^) was also purchased from Sigma-Aldrich. Pea protein isolate (PPI; ≥84 % protein) was supplied by Roquette-Frères SA (Lestrem, France). All other reagents were of analytical grade and used as received. Distilled water was employed throughout the experiments.

### 2.2 Bacterial strain and culture conditions

*Listeria innocua* (ATCC 33090) was used as a surrogate organism. The strain was stored at −80 °C in tryptone soy broth (TSB; Biokar Diagnostics, Pantin, France) supplemented with 20 % (*v*/v) glycerol. Bacterial cultures were prepared according to Liao et al. (2021). One milliliter of thawed stock culture was transferred into 9 mL of TSB and incubated at 37 °C for 8 h. Then, 1 mL of this culture was transferred into 9 mL of fresh TSB and incubated again for 16 h. At the exponential phase, the bacterial suspension was diluted in TSB to approximately 10^6^ CFU/mL for antimicrobial and biofilm assays.

### 2.3 Preparation of pea protein–stabilized oil-in-water nanoemulsions at different pH values

Pea protein isolate (4 g) was dissolved in 5 mM imidazole–acetate buffer adjusted to pH 3.5, 7.0, or 10.0 and stirred overnight at room temperature to ensure complete hydration. The pH was adjusted using 0.1 M or 1.0 M HCl/NaOH as required. Suspensions were centrifuged at 12000 rpm for 20 min to remove insoluble material, and the pH of the supernatant was verified.

Following the protocol of Baghi et al. (2023) with minor modifications, primary coarse emulsions were prepared by adding 20 g of carvacrol to each PPI solution (pH 3.5, 7.0, 10.0) and homogenizing with an Ultra Turrax PT4000 (Kinematica, Switzerland) at 14000 rpm for 5 min. The coarse emulsions were further refined using a Microfluidizer LM20 (Microfluidics Co., MA, USA) with two recirculations at 500 bar and one at 1000 bar. The pH of each final emulsion was readjusted as necessary.

Fresh pepsin or trypsin solutions (0.1 % w/w) were prepared in the same 5 mM buffer at the corresponding pH and added slowly to the fine emulsions under continuous pH control with stirring at 400 rpm. This yielded nine formulations: (i) enzyme-free emulsions, (ii) pepsin-incorporated emulsions, and (iii) trypsin-incorporated emulsions, each prepared at pH 3.5, 7.0, and 10.0. The final composition was 1 % (w/w) PPI, 5 % (w/w) carvacrol, and 0.1 % (w/w) enzyme (when applicable).

### 2.4 Particle size distribution and ζ-potential measurement

The mean hydrodynamic diameter and polydispersity index (PDI) of carvacrol droplets were determined by dynamic light scattering (DLS) using a Zetasizer Nano ZS90 (Malvern Instruments, Worcestershire, UK) at 25 °C and a scattering angle of 90°. Each emulsion (0.1 mL) was diluted in 10 mL of 5 mM imidazole–acetate buffer (pH 3.5, 7.0, or 10.0) to minimize multiple scattering. The refractive indices of carvacrol and water were set to 1.532 and 1.33, respectively, and the absorption coefficient to 0.01. Measurements were recorded after 90 s equilibration, and mean diameters were expressed in nm (*n* = 3).

The ζ-potential was determined by electrophoretic mobility using the same instrument and buffer conditions. Emulsions were suitably diluted to prevent multiple scattering, and each value represented the mean of three measurements. The ζ-potential of pepsin and trypsin solutions (5 mM imidazole–acetate buffer, pH 2–10) was also measured to characterize enzyme charge properties under varying pH.

### 2.5 Microscopic observation of nanoemulsion droplets

A droplet of freshly homogenized emulsion (with or without enzyme) was placed on a clean glass slide, covered with a coverslip, and observed using an EVOS M5000 Imaging Microscope (Thermo Fisher Scientific, USA) at room temperature.

### 2.6 Apparent viscosity measurement

Viscosity was measured using a DV2T viscometer (AMETEK Brookfield, Élancourt, France) equipped with an LV-62 spindle operated at 150 rpm at room temperature. Each sample was analyzed in triplicate.

### 2.7 Creaming stability evaluation

Creaming stability was monitored as an indicator of droplet aggregation or flocculation. Ten milliliters of each freshly prepared nanoemulsion were transferred into transparent glass vials (2 cm × 6 cm) and stored at ambient temperature for 28 days. The heights of the total emulsion (Hₜ) and the serum layer (Hₛ) were measured, and the creaming index (CI) was calculated as:

(1) $$\mathrm{CI}\;\left(\%\right)=\frac{H_{s}}{H_{t}}\times 100$$

### 2.8 Stability of nanoemulsions under different conditions

#### 2.8.1 Ageing stability at room temperature

The effect of enzyme incorporation on droplet stability during storage was examined by monitoring the mean particle size over 28 days at room temperature, following the DLS procedure described in Section 2.4.

#### 2.8.2 Thermal stability at 37 °C

To simulate biofilm treatment conditions, thermal stability was assessed by incubating the emulsions at 37 °C for 0.5, 1, and 2 h. The mean particle size was determined as described above.

### 2.9 Molecular docking simulations

Protein–protein docking simulations were performed to investigate the interactions between enzymes (ligands) and major PPI components, legumin (LEG) and vicilin (VIC) (receptors). Docking was carried out using the HADDOCK 2.4 server (Honorato et al., 2024). The crystal structures of LEG (PDB ID: 3KSC), VIC (7U1I), trypsin (2ZQ1), and pepsin (3PEP) were retrieved from the RCSB Protein Data Bank (https://www.rcsb.org/).

### 2.10 Antibacterial activity against *Listeria innocua*

The minimum inhibitory concentration (MIC) and minimum bactericidal concentration (MBC) of the nanoemulsions were determined using the broth microdilution method in 96-well plates, as described by Chakansin et al. (2022). All emulsions were sterilized by ultraviolet irradiation for 40 min. Two-fold serial dilutions of the nanoemulsions were prepared in pH-adjusted buffer to yield carvacrol concentrations from 0.078 to 5 mg/mL. Each well contained 100 μL of nanoemulsion and 100 μL of L. *innocua* suspension (10^6^ CFU/mL). Sterile buffer and untreated bacterial suspension served as blank and negative controls, respectively. Plates were incubated at 37 °C for 24 h, then 30 μL of 0.015 % (w/v) resazurin solution was added and incubated for 2–4 h. Wells with unchanged blue-violet color were considered above the MIC threshold. For MBC determination, 5 μL from each MIC-positive well was plated on tryptic soy agar (TSA) and incubated for 24 h at 37 °C. The MBC was defined as the lowest concentration yielding no visible colonies. All tests were performed in triplicate.

### 2.11 Antibiofilm activity against *Listeria innocua* biofilm

#### 2.11.1 Biofilm formation assay

Biofilm formation by *L. innocua* was evaluated on polystyrene surfaces over 24, 48, 72, 120, and 168 h at 37 °C using the crystal violet (CV) staining method (Soulaimani et al., 2025). Exponentially growing cultures (10^6^ CFU/mL) were inoculated (200 μL/well) into sterile 96-well microplates and incubated statically. Blank wells contained only sterile TSB. After incubation, wells were gently washed twice with PBS, fixed with 95 % ethanol (250 μL/well, 30 min), air-dried, and stained with 0.1 % (w/v) CV for 45 min in the dark. Excess dye was removed by PBS washing (3–5 times), and the plates were air-dried. Bound dye was solubilized with 95 % ethanol (250 μL/well, 5 min shaking), and absorbance was recorded at 595 nm.

#### 2.11.2 Biofilm eradication assay

The eradication efficiency of enzyme solutions and nanoemulsions against pre-formed *L. innocua* biofilms was assessed using the same CV method, following Soulaimani et al. (2025) with slight modifications. Mature 24 h biofilms were treated with 250 μL of nanoemulsions at MIC, MIC/2, MIC/4, and MIC/8, or equivalent concentrations of pepsin/trypsin in buffer at the corresponding pH. Plates were incubated at 37 °C for 0.5 or 1 h, washed with PBS, and stained with 0.1 % CV for 45 min. Absorbance at 595 nm was measured after ethanol solubilization (10 min).

The biofilm eradication efficiency (EEB) was calculated as:

(2) $$\mathrm{EEB}\;\left(\%\right)=\frac{{\mathrm{OD}}_{\mathrm{negative}}-{\mathrm{OD}}_{\mathrm{sample}}}{{\mathrm{OD}}_{\mathrm{negative}}-{\mathrm{OD}}_{\mathrm{blank}}}\times 100$$

where OD_negative_ is the optical density of formed *L innocua* biofilm treated with buffer; OD_sample_ is the optical density of formed *L innocua* biofilm treated with different samples (nanoemulsions or enzyme solutions); OD_blank_ is the optical density of buffer without biofilm.

#### 2.11.3 Microscopic observation of treated biofilms

Crystal violet-stained biofilms treated with nanoemulsions were imaged at ×10 magnification using the EVOS M5000 microscope (Thermo Invitrogen, USA).

### 2.12 Statistical analysis

All experiments were performed at least in triplicate. Results are expressed as mean ± standard deviation. Statistical significance was determined by one-way analysis of variance (ANOVA) using SPSS software (IBM, v26.0, USA).

## 3 Results and discussion

### 3.1 Physicochemical characterization of enzyme-loaded nanoemulsions at different pH values

#### 3.1.1 Particle size distribution and ζ-potential

**Fig. 1:**
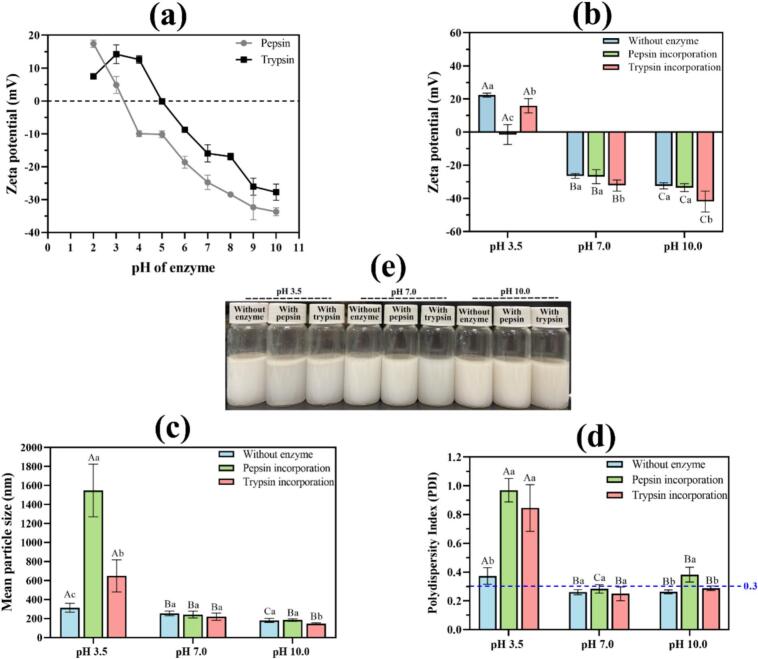
Effect of enzyme incorporation on the ζ-potential, mean droplet size, and polydispersity index (PDI) of pea protein-stabilized carvacrol nanoemulsions at pH 3.5, 7.0, and 10.0. (a) ζ-Potential of pepsin and trypsin solutions measured over the pH range 2.0–10.0; (b–d) ζ-potential, mean droplet diameter, and PDI of pea protein-stabilized nanoemulsions with and without enzyme incorporation at pH 3.5, 7.0, and 10.0; (e) visual appearance of freshly prepared enzyme-loaded nanoemulsions at the three pH conditions. Data are expressed as mean ± SD (*n* = 3). Different lowercase letters indicate statistically significant differences (*p* < 0.05) between enzyme-incorporated and enzyme-free nanoemulsions at the same pH, whereas different uppercase letters denote significant differences among nanoemulsions prepared at different pH values within the same formulation type.

**Fig. 2:**
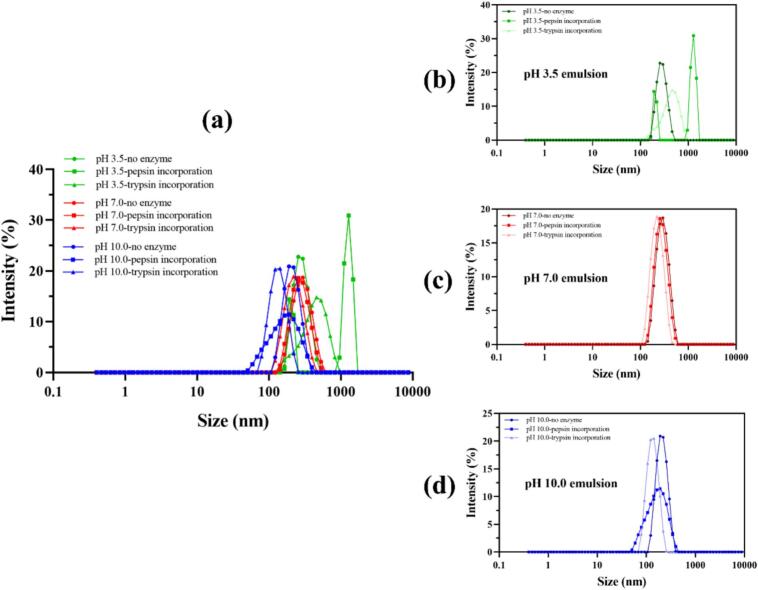
Effect of enzyme incorporation on the particle size distribution of pea protein–stabilized carvacrol nanoemulsions at pH 3.5, 7.0, and 10.0. (a) Overlay of size distribution curves for the nine nanoemulsion formulations combining all pH conditions; (b–d) individual particle size distributions of nanoemulsions prepared at pH 3.5, 7.0, and 10.0, respectively, with and without incorporation of pepsin or trypsin. All distributions were obtained by dynamic light scattering (DLS) at 25 °C after appropriate dilution in 5 mM imidazole–acetate buffer adjusted to the corresponding pH.

Nine formulations were prepared by incorporating pepsin or trypsin (0.1 % *w*/w) into pre-formed PPI-stabilized carvacrol nanoemulsions (1 % w/w PPI; 5 % w/w oil) at pH 3.5, 7.0, and 10.0 (Fig. 1e). All original emulsions displayed submicron droplets (≈180–314 nm) with monomodal distributions and low PDI (Figs. 1c, 2), in line with prior work on plant-protein emulsifiers (Baghi et al., 2023; Burger & Zhang, 2019). As expected, ζ-potential magnitudes were higher at pH 7.0 and 10.0 than near the isoelectric region, consistent with smaller droplets and improved emulsification at pH values further from the protein's pI (Fig. 1b; Burger & Zhang, 2019).

**Fig. 3:**
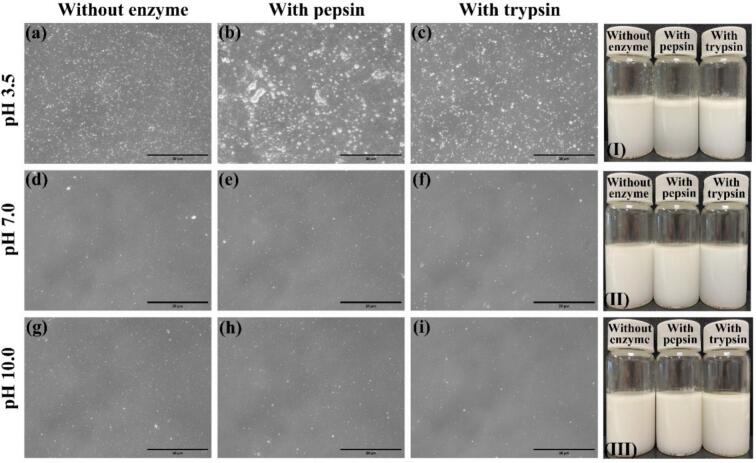
Microscopic observation and visual appearance of pea protein–stabilized carvacrol nanoemulsions with and without enzyme incorporation at different pH values. (a–c) Optical micrographs of nanoemulsions at pH 3.5; (d–f) at pH 7.0; (g–i) at pH 10.0, showing formulations without enzyme, with pepsin, and with trypsin, respectively. White dots correspond to oil droplets within the nanoemulsion. Scale bars = 20 μm. (I–III) Visual appearance of freshly prepared nanoemulsions containing pepsin or trypsin at pH 3.5 (I), 7.0 (II), and 10.0 (III).

Enzyme charge profiling revealed distinct pH-dependent behaviors for pepsin and trypsin. At pH 3.5, pepsin exhibited a weakly negative to nearly neutral charge, whereas trypsin was predominantly cationic. In contrast, both enzymes displayed strongly negative charges in the pH range of 7.0–10.0 (Fig. 1a). The addition of pepsin at neutral and alkaline pH values resulted in negligible changes in ζ-potential, mean droplet size, and polydispersity index (PDI) (Fig. 1, Fig. 2). This observation aligns with the known inactivation of pepsin above pH 6.0 and its consequently limited capacity to alter interfacial properties under these conditions (Heda et al., 2023). In contrast, trypsin incorporation increased the absolute ζ-potential at pH 7.0 (−26.5 → −32.2 mV) and pH 10.0 (−32.4 → −41.9 mV), concomitant with reductions in mean size and PDI, most notably to ≈145 nm at pH 10.0 (Figs. 1c–d, 2d), indicating stronger electrostatic repulsion and enhanced colloidal stability. This trend agrees with reports where moderate tryptic hydrolysis generates surface-active peptides that improve interfacial packing and droplet refinement in plant-protein systems (Xu et al., 2025). Microscopic observations further supported these findings, revealing increased inter-droplet distances in emulsions treated with trypsin (Fig. 3f, i).

At pH 3.5, original emulsions showed larger droplets, higher PDI, and lower |ζ| than at pH 7.0–10.0, reflecting reduced stability near pI. Under these acidic conditions, pepsin induced severe destabilization with near-neutral ζ, micron-scale droplets (≈1.55 μm), and PDI approaching 1.0 (Fig. 1, Fig. 2, Fig. 3b). The result is consistent with strong peptic activity and interfacial film erosion at low pH (Khan et al., 2022; Salelles et al., 2021; Zhang et al., 2021). Trypsin at pH 3.5 also impaired stability, increasing size and PDI and lowering |ζ| (Fig. 1, Fig. 2, Fig. 3c), which aligns with limited activity and charge screening that perturb the interfacial layer in acidic media (Wang, Liu, et al., 2022).

#### 3.1.2 Apparent viscosity behavior

**Fig. 4:**
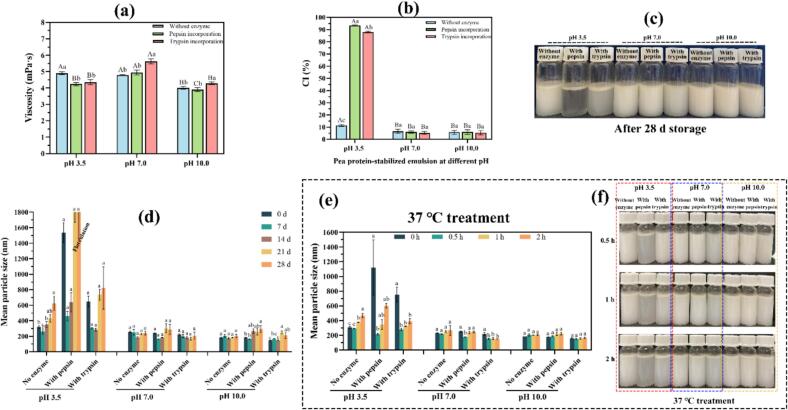
Effect of enzyme incorporation on the rheological and stability properties of pea protein–stabilized carvacrol nanoemulsions at different pH values. (a) Apparent viscosity of freshly prepared nanoemulsions at pH 3.5, 7.0, and 10.0; (b–d) variation of creaming index, visual appearance, and mean droplet size during storage at room temperature for 4 weeks; (e–f) variation of mean droplet size and visual appearance after incubation at 37 °C for 0.5, 1, and 2 h. In (a) and (b), different lowercase letters indicate statistically significant differences (*p* < 0.05) between enzyme-incorporated and enzyme-free nanoemulsions at the same pH, while different uppercase letters denote significant differences among nanoemulsions prepared at different pH values within the same formulation type. In (d) and (e), lowercase letters indicate statistically significant differences among the same nanoemulsion formulation at different storage durations (d) or incubation times at 37 °C (e).

Apparent viscosity depended on pH and enzyme treatment (Fig. 4a). Emulsions at pH 10.0 exhibited the lowest values (≈3.90–4.29 mPa·s), whereas higher viscosities were recorded at pH 3.5 and 7.0 (≈4.23–5.63 mPa·s). These observations are consistent with pH-modulated protein conformation and aggregation that alter droplet interactions and continuous-phase structuring (Hu et al., 2016; Li et al., 2022). At pH 7.0–10.0, trypsin increased viscosity relative to the original emulsions, likely due to moderate interfacial hydrolysis that generates adsorbing peptides and, when in excess, promotes reversible depletion or weak bridging between droplets, thereby increasing bulk viscosity and stability (Olsmats et al., 2025). At pH 3.5, trypsin addition decreased viscosity, consistent with charge perturbation without effective interfacial reinforcement. Pepsin produced negligible changes at pH 7.0–10.0 but reduced viscosity at pH 3.5, in agreement with interfacial film deterioration under shear when pepsin is active (Wang, Wang, et al., 2022). Overall, these rheological results are consistent with the DLS and ζ-potential trends observed (Fig. 1, Fig. 2).

#### 3.1.3 Droplet morphology and creaming stability during storage

After 28 days of storage at room temperature, nanoemulsions prepared at pH 7.0–10.0 exhibited no visible serum layer and showed a very low creaming index (CI < 5 %), with mean droplet diameters remaining below approximately 300 nm (Fig. 4b–d), consistent with strong repulsion and limited coalescence (Liao et al., 2022). In contrast, all enzyme-treated emulsions at pH 3.5 exhibited pronounced phase separation (high CI), especially with pepsin where CI exceeded 90 %, indicative of extensive interfacial degradation and irreversible coalescence. Notably, the transient decreases in apparent size during early storage can be attributed to breakup of weak flocs upon dilution or stirring, followed by growth dominated by coalescence and Ostwald ripening (Chutinara et al., 2024; Liao et al., 2021). Thermal challenges at 37 °C up to 2 h did not compromise neutral or alkaline emulsions (Fig. 4e–f). Notably, at pH 7.0 the trypsin-treated emulsion showed further size reduction, consistent with closer-to-optimal tryptic activity at 37 °C that produces highly surface-active peptides and strengthens the interfacial film. At pH 3.5, pepsin activity accelerated interfacial degradation; peptide accumulation transiently promoted depletion flocculation that was disrupted upon dilution, before coarsening became dominant with time (Shahbazi et al., 2024).

### 3.2 Molecular docking insights into enzyme–emulsion interfacial interactions

**Fig. 5:**
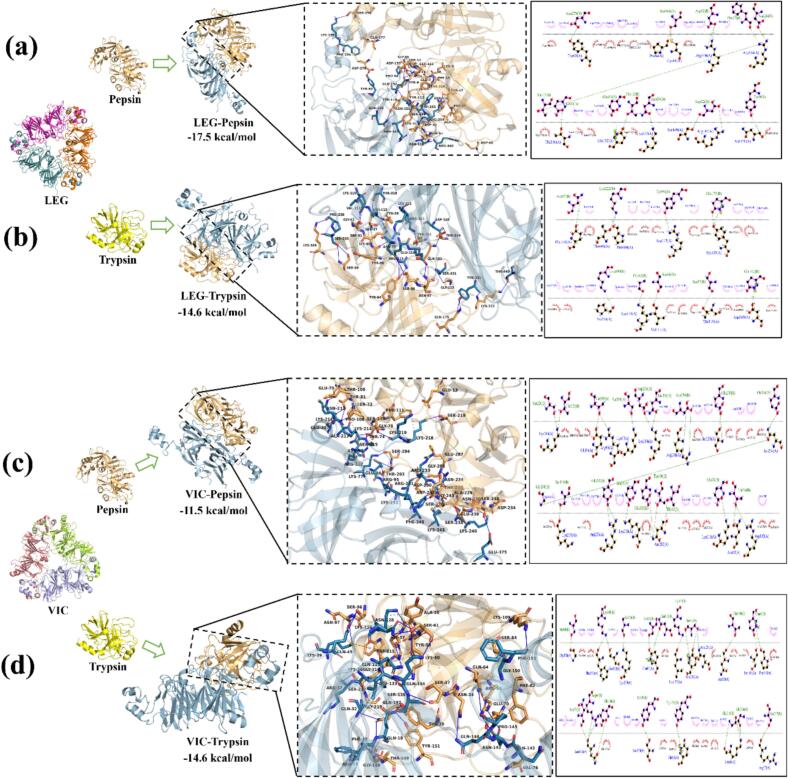
Predicted binding interactions between pepsin or trypsin and the major pea protein components legumin (LEG) and vicilin (VIC) obtained by molecular docking. (a) LEG–Pepsin, (b) LEG–Trypsin, (c) VIC–Pepsin, and (d) VIC–Trypsin complexes showing optimal docking conformations. The visualized interactions highlight hydrogen bonding and hydrophobic contacts at the enzyme–substrate interfaces, supporting the experimentally observed pH-dependent effects on emulsion stability and bioactivity.

Protein–protein docking was performed between pepsin or trypsin and the major PPI globulins legumin (LEG) and vicilin (VIC) (Burger & Zhang, 2019). All complexes displayed extended interfaces with numerous hydrogen bonds and hydrophobic contacts, indicating strong interactions. Predicted binding free energies were −17.5 (LEG–Pepsin), −14.6 (LEG–Trypsin), −11.5 (VIC–Pepsin), and −14.6 kcal mol^−1^ (VIC–Trypsin) (Fig. 5).

For pepsin, LEG–Pepsin exhibited stronger binding than VIC–Pepsin, with interaction maps revealing multiple hydrogen bonds and salt bridges. A high participation of charged residues (Arg/Lys/Asp/Glu) on LEG/VIC in pepsin binding helps rationalize the near-neutralization of droplet charge and the severe instability observed at pH 3.5. The involvement of acidic residues near the catalytic dyad (Asp32/Asp215) in interface binding could also modulate catalytic efficiency and interfacial residence, thereby intensifying interfacial proteolysis under acidic conditions (Zhao et al., 2022). The overall picture is consistent with the marked deterioration of stability upon pepsin addition at pH 3.5 and the limited impact at pH 7.0–10.0 where pepsin is inactive.

For trypsin, LEG–Trypsin and VIC–Trypsin showed similar binding energies, mainly through hydrogen bonding and hydrophobic contacts. Interaction patterns reflected recognition of Lys/Arg-rich regions by the anionic S1 pocket, including π-cation interactions, while residues from the catalytic triad contributed little to docking, suggesting predominant non-productive binding or limited cleavage at the interface (Sneha et al., 2022). Such partial hydrolysis can yield short amphiphilic peptides that improve interfacial packing and increase |ζ|, in agreement with the physicochemical data at pH 7.0–10.0.

### 3.3 Enhancement of antimicrobial and antibiofilm activities by enzyme incorporation

#### 3.3.1 Antibacterial efficacy against *L. innocua*

MIC and MBC against planktonic *L. innocua* are summarized in Table 1. Original nanoemulsions exhibited MIC = 312.5 μg/mL at all pH values, in agreement with previous reports on carvacrol nanoencapsulation (Yammine, Gharsallaoui, Fadel, Karam, et al., 2023). The anti-*L. innocua* activities of trypsin and pepsin showed no noticeable effects, and MIC and MBC values were not applicable (data not shown). At pH 7.0–10.0, enzyme loading did not change MIC, consistent with the fact that pepsin and trypsin are not intrinsically bactericidal (Mechmechani et al., 2023). At pH 3.5, pepsin increased MIC to 625 μg/mL, plausibly due to instability, premature release, or heterogenous distribution of carvacrol. Under alkaline conditions, MBC equaled MIC, which is consistent with smaller droplets and improved dispersion that favor membrane interaction and diffusion of carvacrol; at other pH, MBC tended to rise to 625 μg/mL, consistent with larger droplet size and reduced effective surface area, as noted by Yammine, Gharsallaoui, Fadel, Mechmechani, et al. (2023).

**Table 1:** Effects of enzyme incorporation on the minimum inhibitory concentration (MIC) and the minimum bactericidal concentration (MBC) of pea protein–stabilized carvacrol nanoemulsions at pH 3.5, 7.0, and 10.0 against *Listeria innocua*.

Pea protein-stabilized carvacrol nanoemulsions		Without enzyme	With pepsin	With trypsin
pH 3.5	MIC (μg/mL)	312.5	625	312.5
	MBC (μg/mL)	312.5	625	625

pH 7.0	MIC (μg/mL)	312.5	312.5	312.5
	MBC (μg/mL)	625	625	625

pH 10.0	MIC (μg/mL)	312.5	312.5	312.5
	MBC (μg/mL)	312.5	312.5	312.5

Note: All concentration values represent the concentration of carvacrol in the nanoemulsions.

#### 3.3.2 Antibiofilm effects on pre-formed *L. innocua* biofilms

**Fig. 6:**
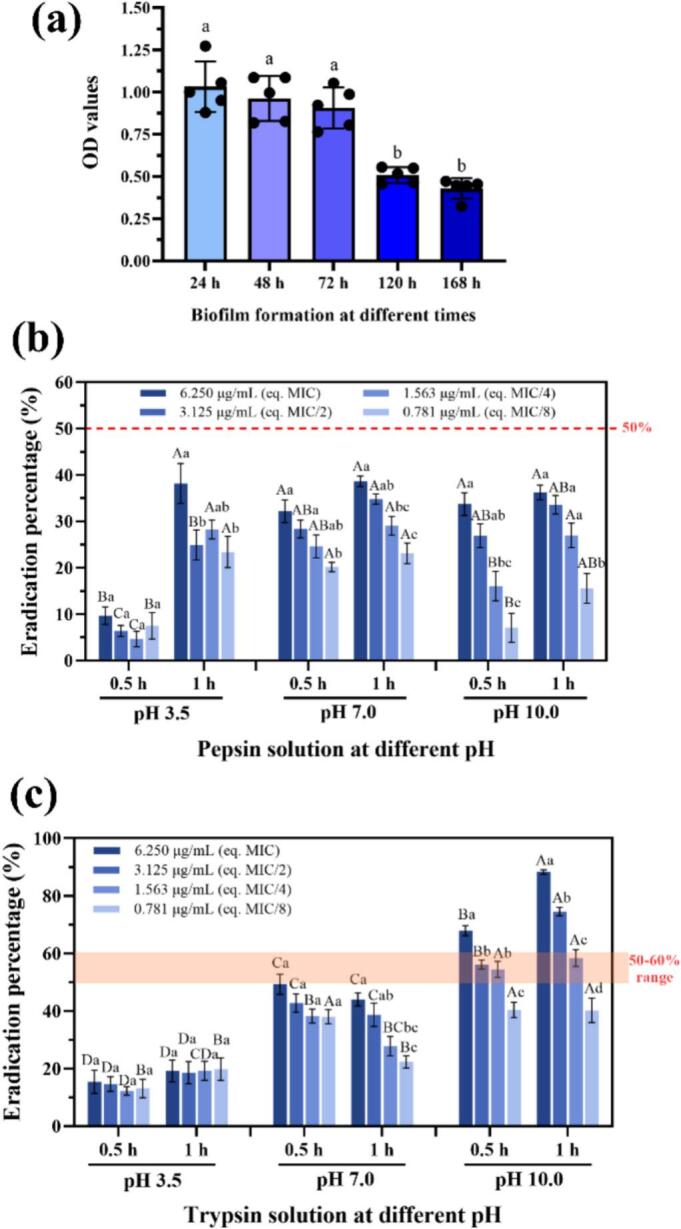
Biofilm formation and enzymatic eradication efficiency of *Listeria innocua* on polystyrene surfaces. (a) Biofilm formation of *L. innocua* on 96-well polystyrene microplates after 24, 48, 72, 120, and 168 h of static incubation at 37 °C; different lowercase letters indicate statistically significant differences (p < 0.05) among incubation times. (b–c) Effects of pepsin and trypsin solutions at various concentrations and pH values on the eradication of preformed L. *innocua* biofilms after 0.5 h and 1 h of treatment. Enzyme concentrations were equivalent (eq.) to those present in the nanoemulsions at MIC, MIC/2, MIC/4, and MIC/8 levels. Lowercase letters indicate statistically significant differences (p < 0.05) among enzyme concentrations at the same pH and treatment time, while uppercase letters denote significant differences between treatment times for enzyme solutions of the same concentration across different pH conditions.

Biofilm development on polystyrene peaked within the first three days at 37 °C and then declined, likely due to nutrient limitation and detachment events (Fig. 6a; Xue & Kang, 2025). A 24 h maturation period was therefore selected for eradication assays.

Enzyme-only treatments showed modest performance overall. Pepsin removal remained below 50 % across conditions, whereas trypsin exceeded 50 % only at pH 10.0, with clear dose- and time-dependencies at pH 7.0–10.0 (Fig. 6b–c). The higher efficacy of trypsin at neutral and alkaline pH is consistent with Lys/Arg-specific hydrolysis of proteinaceous EPS domains, which facilitates detachment (Wang et al., 2023). Limited effects at pH 3.5 (EEB = 12–20 %) can be attributed to trypsin inactivation and to matrix compaction at low pH that impairs penetration (Azeem et al., 2025; Ikäläinen et al., 2024).

**Fig. 7:**
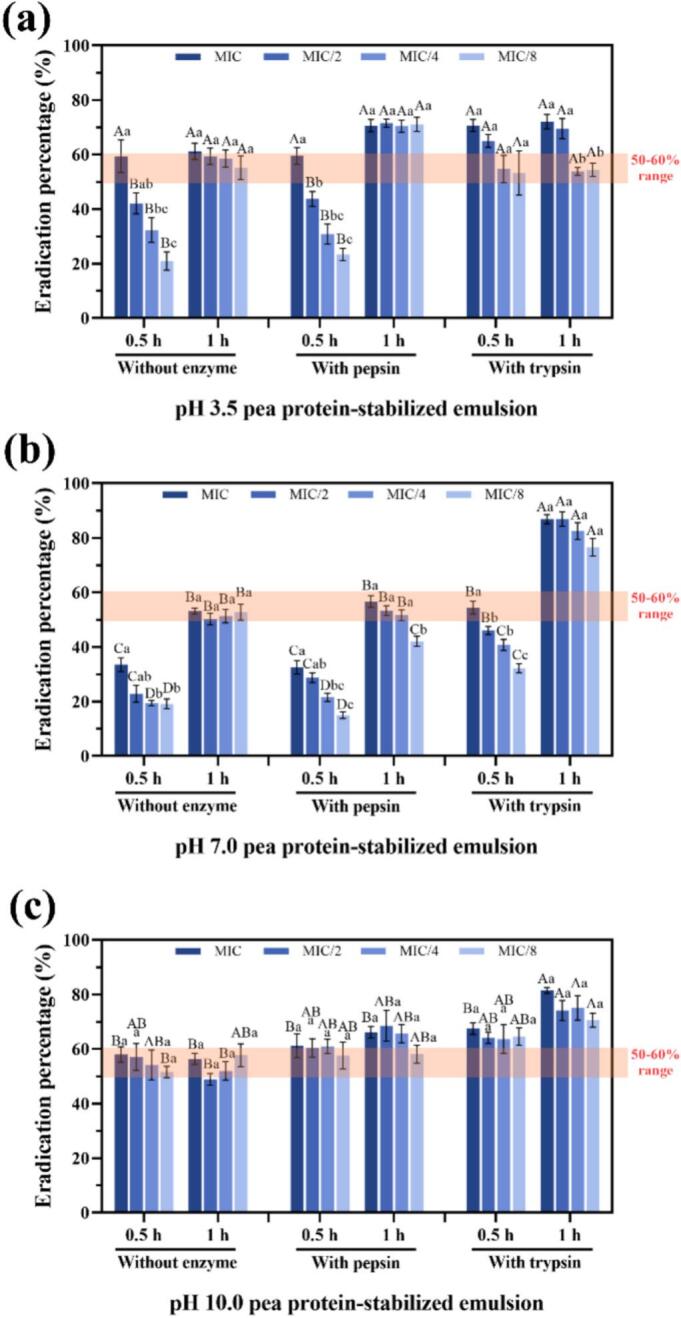
Effect of enzyme incorporation on the eradication of *Listeria innocua* biofilms by pea protein–stabilized carvacrol nanoemulsions at different pH values. (a–c) Eradication efficiency of *L. innocua* biofilms formed on polystyrene surfaces after 0.5 h and 1 h of treatment with nanoemulsions at pH 3.5 (a), 7.0 (b), and 10.0 (c) using different concentrations corresponding to MIC, MIC/2, MIC/4, and MIC/8. Different lowercase letters indicate statistically significant differences (p < 0.05) among MIC levels for the same nanoemulsion type and treatment time. Different uppercase letters indicate statistically significant differences (p < 0.05) between nanoemulsion types at the same MIC level across treatment times.

**Fig. 8:**
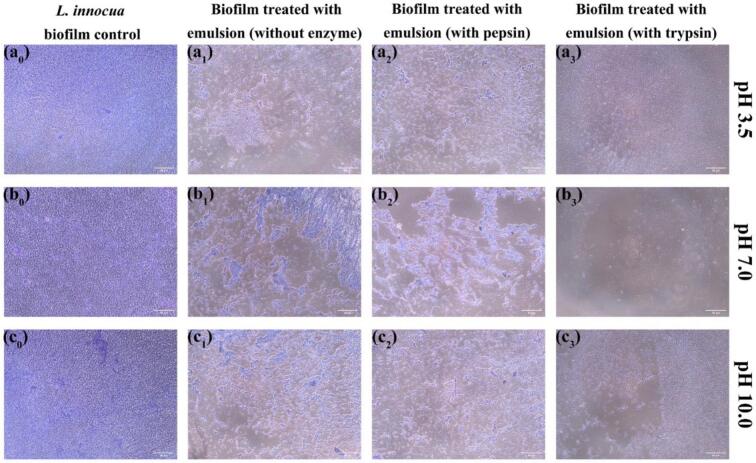
Microscopic observation of *Listeria innocua* biofilms after treatment with enzyme-loaded pea protein–stabilized carvacrol nanoemulsions. Representative optical micrographs of *L. innocua* biofilms formed on polystyrene surfaces after 1 h of exposure to nanoemulsions (MIC concentration) at pH 3.5, 7.0, and 10.0, with and without incorporation of pepsin or trypsin. Scale bars = 50 μm.

Nanoemulsions showed rapid, high-level eradication at pH 10.0, frequently surpassing 50 % within 0.5 h regardless of concentration, which is consistent with a more open, permeable matrix in alkaline environments. Trypsin-loaded emulsions outperformed pepsin-loaded and enzyme-free systems (increasing by approximately 16 % and 25 %, respectively), reaching ≈67–82 % eradication at MIC within 0.5–1 h, in line with the trypsin-solution trend (Fig. 7; Fig. 8). At pH 7.0, all emulsions displayed clear dose- and time-responses; the pepsin-loaded emulsions showed similar performance to the enzyme-free controls, with maximum biofilm eradication achieved at the MIC after 1 h, reaching 57 % and 53 %, respectively. However, the trypsin-loaded emulsions reached ≈77–87 % after 1 h, which was approximately 30 % higher than that of trypsin alone (at low concentrations) and also around 30 % higher than the enzyme-free group, supporting a synergy between active trypsin and carvacrol delivery. These observations are consonant with sequential enzyme–carvacrol synergies reported for *P. aeruginosa* and *E. faecalis* (Mechmechani et al., 2022, Mechmechani et al., 2023). At pH 3.5, all emulsions retained good antibiofilm activity and outperformed enzyme-only treatments; trypsin gave a slight additional gain (approximately 10 % higher than the enzyme-free group), plausibly by locally altering the interfacial layer and facilitating faster carvacrol release. Interestingly, in spite of the physical instability of pepsin-treated emulsions at pH 3.5, antibiofilm efficacy remained high, achieving over 70 % eradication rate across all concentrations within 1 h, which were approximately 10–15 % higher than the enzyme-free group. This indicates that rapid carvacrol availability and diffusion can compensate for colloidal destabilization during the short exposure times used.

Taken together, the pH-dependent gains in eradication, particularly with trypsin at pH 7.0–10.0, suggest that enzyme–interface remodeling and matrix permeability jointly condition carvacrol delivery, thereby motivating the mechanistic analysis below (Section 3.4).

### 3.4 Proposed mechanism of stabilization and bioactivity enhancement

The combined experimental and in silico data support a mechanistic model that links pH-dependent enzyme–protein interactions at the oil–water interface to colloidal stability and bioactivity:

***Neutral and alkaline media (pH 7.0–10.0).*** Trypsin interacts preferentially with Lys/Arg-rich motifs on LEG/VIC. Docking suggests mainly non-productive binding with limited cleavage near the catalytic triad, which favors moderate interfacial proteolysis. The resulting short amphiphilic peptides adsorb rapidly, tighten interfacial packing, and increase the magnitude of ζ-potential through selective sequestration of cationic residues, leading to smaller droplets, higher |ζ|, and lower creaming. Under biofilm conditions, active trypsin simultaneously weakens proteinaceous EPS domains, increasing matrix permeability. The more robust, peptide-reinforced interface promotes sustained yet bioavailable carvacrol presentation, and the more open EPS facilitates its diffusion and membrane disruption, producing superior antibiofilm effects.

***Acidic media (pH 3.5).*** Pepsin binds strongly to LEG/VIC, involving numerous charged residues and acidic side chains that align with catalytic motifs. This promotes extensive interfacial proteolysis, erosion of the protein film, charge neutralization, and aggregation or coalescence, consistent with high CI and large sizes. Despite colloidal destabilization, rapid release and high local concentration of carvacrol can still yield effective antibiofilm action over short exposures. Trypsin is largely inactive, contributes little beneficial hydrolysis, and can perturb interfacial electrostatics, which explains the modest decrease in stability without major gains in activity.

***Matrix-level consequences.*** At alkaline pH, EPS deprotonation reduces cation bridging and opens the biofilm network, which facilitates penetration of enzymes and nanoemulsion droplets and lowers the dose–time requirement for eradication. At acidic pH, EPS compaction hampers enzyme diffusion; antibiofilm efficacy is then dominated by fast-acting carvacrol rather than by proteolysis.

Overall, trypsin-enabled peptide remodeling of the interface under neutral/alkaline conditions explains the concurrent gains in physical stability (smaller droplets, higher |ζ|, low CI) and biological performance (higher eradication), whereas pepsin-driven interfacial erosion at acidic pH accounts for instability yet does not preclude strong, carvacrol-mediated antibiofilm effects.

## 4 Conclusion

This study demonstrated that the incorporation of proteolytic enzymes markedly influenced the physicochemical stability and biological functionality of pea protein–stabilized carvacrol nanoemulsions in a pH-dependent manner. Under neutral and alkaline conditions, nanoemulsions exhibited small droplet sizes (≈ 254 nm and 180 nm), high absolute ζ-potentials (−26.5 mV and −32.4 mV), and excellent storage stability over four weeks (CI < 5 %). Trypsin further improved emulsification efficiency through moderate interfacial hydrolysis that generated surface-active peptides and strengthened the interfacial film. Conversely, pepsin induced marked destabilization at acidic pH through extensive proteolysis and charge neutralization, while remaining inactive and harmless to emulsion structure at higher pH. The antibacterial activity against *Listeria innocua* planktonic cells was governed primarily by carvacrol, with a consistent MIC of 312.5 μg/mL across conditions except under acidic pH, where pepsin-induced instability reduced efficacy. In contrast, antibiofilm performance depended strongly on enzyme activity: trypsin produced a pronounced synergistic effect with carvacrol at pH 7.0–10.0, achieving substantial biofilm eradication, whereas pepsin conferred only minor benefits under acidic conditions. Molecular-docking analyses clarified these observations at the molecular scale. Both legumin and vicilin displayed strong affinity for the enzymes via extensive hydrogen bonding and hydrophobic contacts. Pepsin formed multiple salt bridges involving charged Arg/Lys and Asp/Glu residues, which likely disrupted the interfacial protein layer and contributed to instability. Trypsin, by contrast, bound Lys/Arg residues in a predominantly non-catalytic mode, enhancing negative surface charge and promoting interfacial stabilization.

Overall, these findings highlight trypsin-based PPI nanoemulsions as promising multifunctional antimicrobial and antibiofilm systems, while underscoring the importance of controlling pH-dependent enzyme incorporation to achieve optimal balance between colloidal stability and biological performance for food and biomedical sanitation applications.

## CRediT authorship contribution statement

**Jun Ji:** Writing – original draft, Methodology, Formal analysis, Data curation. **Mohamed Brahmi:** Writing – review & editing. **Emilie Dumas:** Visualization, Supervision, Investigation. **Nour-Eddine Chihib:** Validation, Supervision, Conceptualization. **Adem Gharsallaoui:** Writing – review & editing, Validation, Supervision, Project administration, Methodology.

## Declaration of competing interest

The authors declare that they have no known competing financial interests or personal relationships that could have appeared to influence the work reported in this paper.

## Data Availability

Data will be made available on request.
